# Supplementary figures and images for: Metaplastic sleep regulation in Drosophila determined by microscale circadian neural dynamics
Source: bioRxiv. 2026 Mar 24:2026.03.21.713346. Preprint. [Version 1] doi: 10.64898/2026.03.21.713346 (PMC13042052; doi:10.64898/2026.03.21.713346)

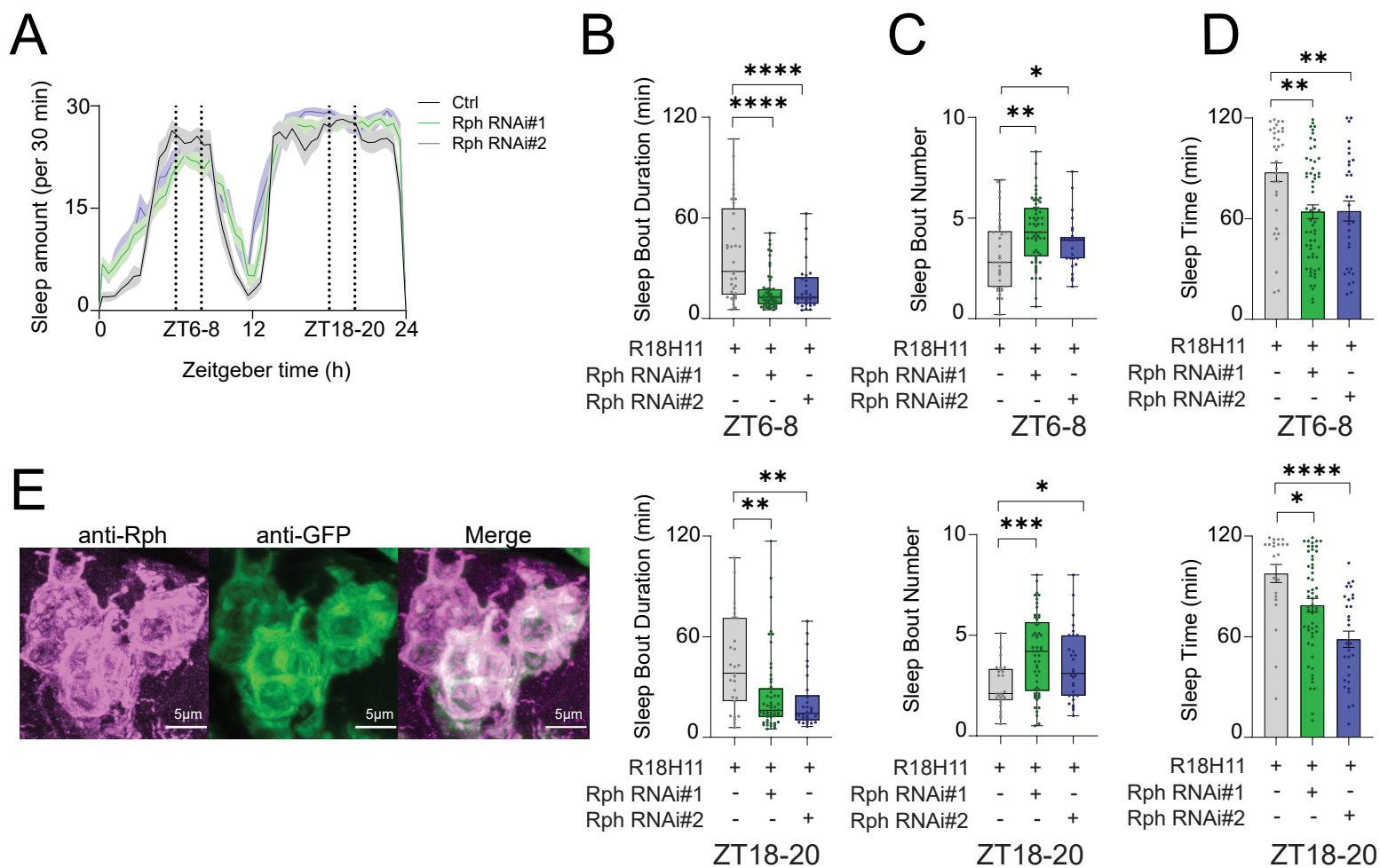

Fig. S1.

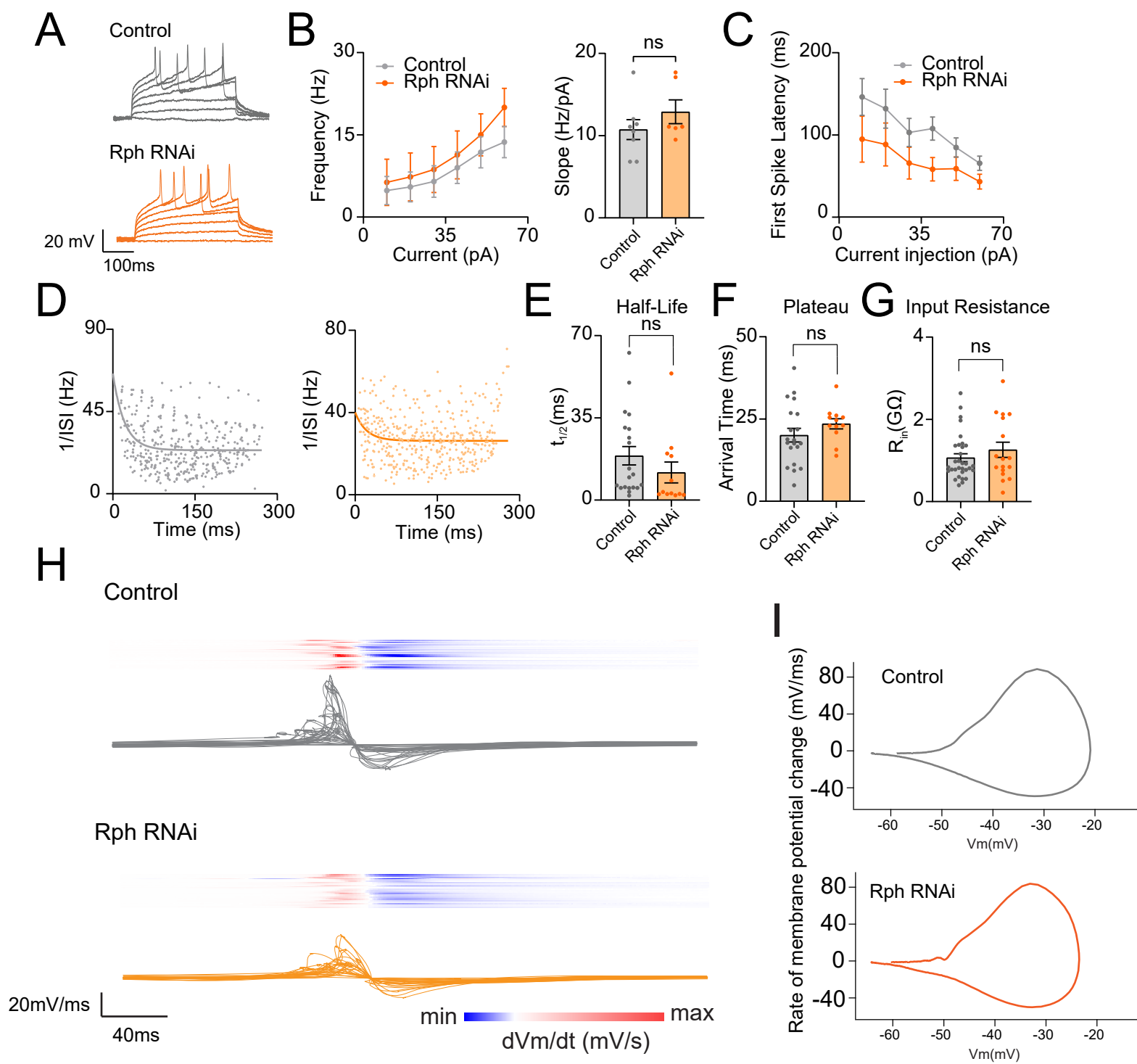

Fig. S2.

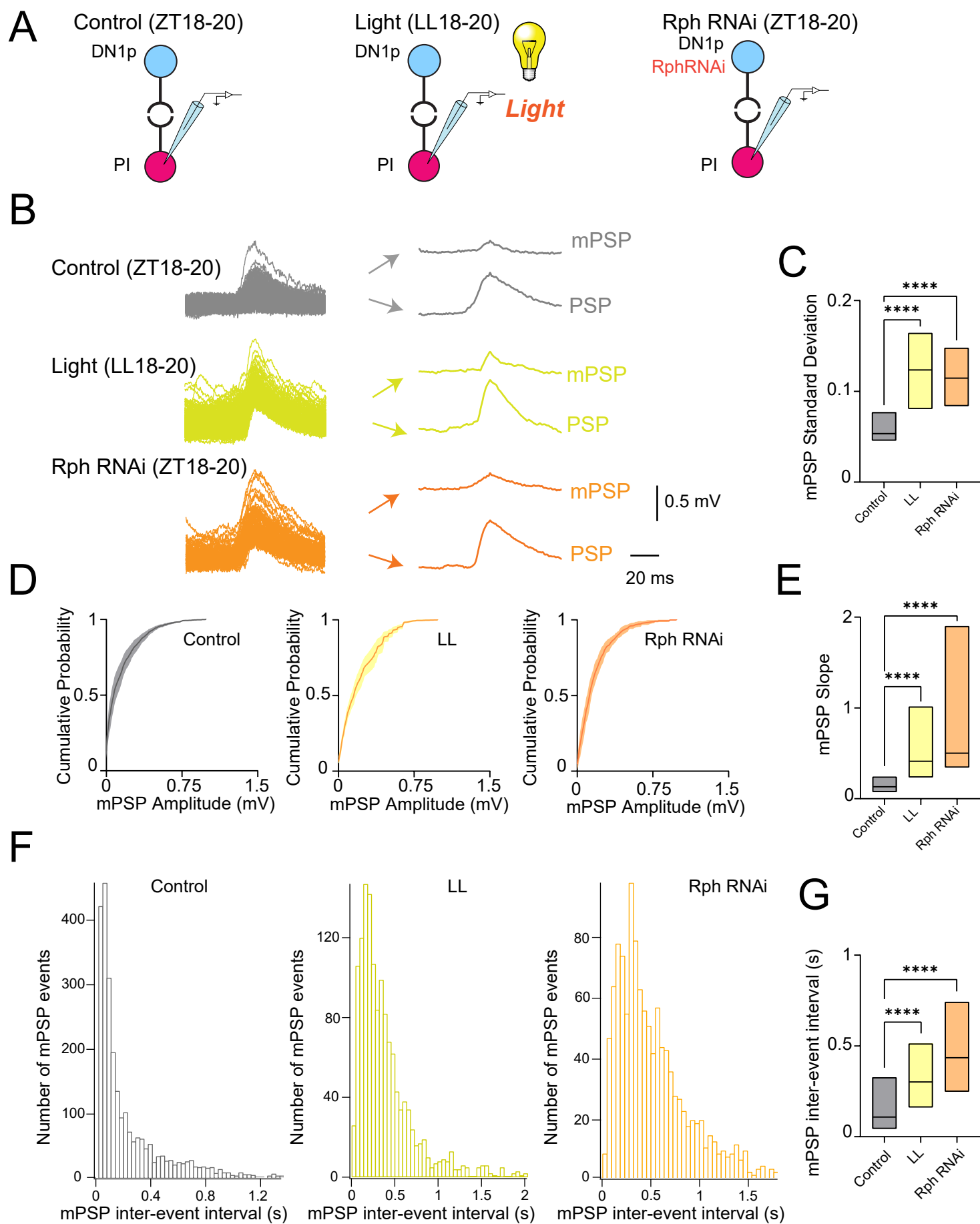

Fig. S3.

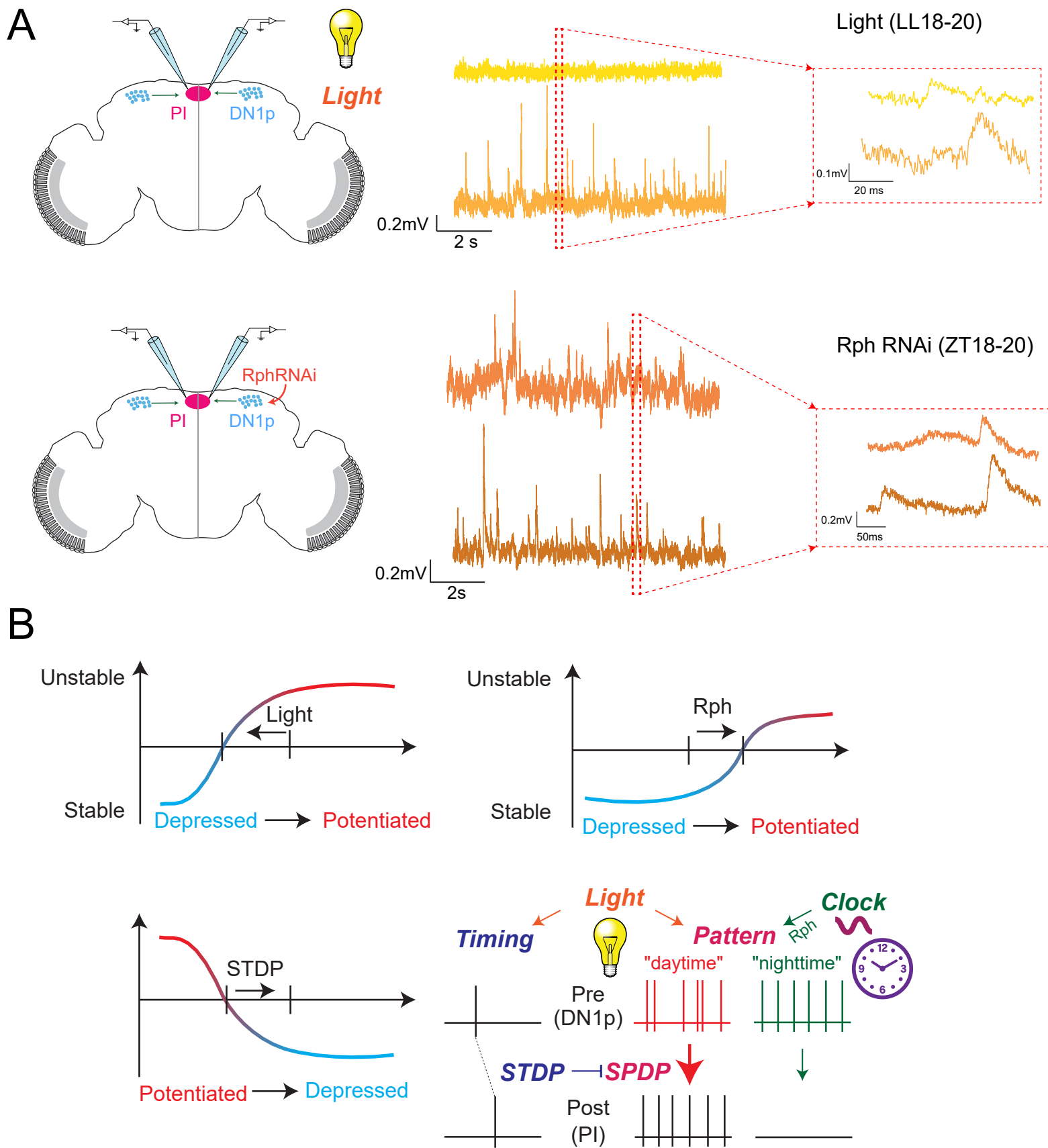

Fig. S4.

Supplement: Supplement 1 — Supplementary Fig. 1 Additional sleep phenotypes and immunostaining of Rph in DN1p neurons. (A) Twenty-four-hour sleep profiles of R18H11-GAL4 flies (gray, N = 32), R18H11-GAL4>UAS-Rph RNAi #1 flies (green, N = 32), and R18H11-GAL4>UAS-Rph RNAi #2 flies (blue, N = 32). (B–D) Quantification of sleep parameters during ZT6–8 and ZT18–20 for the genotypes shown in A. (B) Sleep-bout duration. (C) Sleep-bout number. (D) Total sleep time. (E) Immunohistochemistry showing Rph in DN1p cell bodies using anti-Rph (Rabphilin 3A) (magenta) and anti-GFP (green) labeling. **p < 0.01, ***p < 0.001, ****p < 0.0001. ns: non-significance based on one-way ANOVA followed by post-hoc Tukey tests. Supplementary Fig. 2 Additional electrophysiological analyses of DN1p firing and intrinsic membrane properties assessed by current injection. (A) Representative membrane-potential traces showing current-evoked firing in control flies (gray) and flies expressing Rph RNAi in DN1p neurons (orange). (B) Firing rate–current (f–I) relationship for control flies (N = 8) and flies expressing Rph RNAi in DN1p neurons (N = 6) at ZT18–20; slope factors from linear regression are compared. (C) First-spike latency as a function of injected current in control flies and flies expressing Rph RNAi in DN1p neurons at ZT18–20. (D) Boltzmann fits to the time course of reciprocal interspike interval (1/ISI) during current-evoked firing in control flies and flies expressing Rph RNAi in DN1p neurons at ZT18–20. (E) Half-time derived from the fits shown in D (control, N = 20; Rph RNAi, N = 12). (F) Plateau-arrival time derived from the fits shown in D (control, N = 20; Rph RNAi, N = 12). (G) Input resistance measured from membrane-voltage responses to current injection at ZT18–20 (control, N = 32; Rph RNAi, N = 17). (H) Distribution of spike-onset velocity values from spontaneous action potentials, with superimposed example waveforms for control flies and flies expressing Rph RNAi in DN1p neurons at ZT18–20. (I) Phase [file media-1.pdf]
